# The Evolution and Impact of Mixed Mood States: From Ancient Observations to Modern Understanding

**DOI:** 10.1192/j.eurpsy.2025.787

**Published:** 2025-08-26

**Authors:** V. I. Grasso, D. G. Sotelo, M. Cetkovich, M. Dines, B. Hofmann, G. Vazquez

**Affiliations:** 1 psychiatry, CIPCO; 2 psychiatry, private practice, Cordoba; 3 psychiatry, INECO, Buenos aires, Argentina; 4 psychiatry, private practice, Palma de mallorca, Spain; 5 psychiatry, Providance Care Hospital, kingston, Canada

## Abstract

**Introduction:**

Mood disorders with mixed features, characterized by the concurrent presence of manic and depressive symptoms, represent a complex and significant area in psychiatric research. Historically, the recognition of such mixed states traces back to ancient Greek scholars, with evolving understanding through the 19th century. This evolution reflects substantial contributions from early thinkers to modern psychiatric frameworks.

**Objectives:**

1. To trace the historical development of the concept of mixed mood states from ancient Greece to the 19th century.
2. To analyze the contributions of key figures such as Areteo of Cappadocia, Wilhelm Griesinger, Jean-Pierre Falret, Emil Kraepelin, and Wilhelm Weygandt in shaping the understanding of mixed states.
3. To evaluate the impact of Kraepelin’s and Weygandt’s work on contemporary classifications of mood disorders.

**Methods:**

A historical review of primary and secondary sources, including classical texts and psychiatric literature, was conducted. The analysis focused on the contributions of significant historical figures, examining their theories and classifications of mood disorders. Key publications, such as Kraepelin’s treatises and Weygandt’s monographs, were scrutinized to assess their influence on modern psychiatric nomenclature.

**Results:**

The study highlights the foundational observations of mixed mood states by ancient Greek scholars, particularly Areteo, who first proposed that mania and melancholia were facets of the same condition. The 19th-century revival of interest in these states saw important contributions from Griesinger and Falret, with Kraepelin’s systematic framework in his 1899 treatise integrating mixed states into a unified concept of manic-depressive insanity. Weygandt’s 1899 monograph further refined the understanding of mixed states, reflecting a collaborative intellectual effort with Kraepelin.

**Conclusions:**

The historical evolution of mixed mood states demonstrates a significant advancement in psychiatric theory. Kraepelin’s comprehensive framework, supported by Weygandt’s detailed analysis, laid the groundwork for contemporary classifications of mood disorders. Recognizing the collaborative nature of these developments underscores the importance of shared intellectual contributions in the advancement of psychiatric science.

**Disclosure of Interest:**

None Declared

